# ImagiChem: Hybrid Deterministic Image-Conditioned Generation of Chemically Valid and Drug-like Molecules from Artistic Inputs

**DOI:** 10.1021/acsomega.6c05429

**Published:** 2026-07-09

**Authors:** Rocco Buccheri, Antonio Rescifina

**Affiliations:** Department of Drug and Health Sciences, 9298 University of Catania, Viale A. Doria 6, 95125 Catania, Italy

## Abstract

Generative artificial intelligence (AI) has redefined the exploration and expansion of the chemical space. However, the systematic use of visual information as a chemical prior remains virtually unexplored. Herein, we introduce ImagiChem, a deterministic image-conditioned generative framework that transforms visual patterns into chemically valid and drug-like molecules via three complementary generation modes: a library-based engine that assembles molecules from curated pharmacophore cores guided by pixel statistics, a rule-based from-scratch generator whose structural blueprint is governed by the global image profile, and a hybrid mode that combines both engines and merges their outputs. By encoding local color distributions, gradient statistics, and texture descriptors, as well as global image features, such as spatial coherence, chromatic harmony, and morphological class, into an alphabet of atomic, functional, and topological rules, ImagiChem establishes a direct multimodal mapping between visual features and molecular connectivity. This approach ensures compliance with physicochemical constraints, such as the Lipinski and Veber criteria, low pan-assay interference compounds (PAINS) incidence, and synthetic accessibility (SA), while yielding over 99% novel structures absent from current chemical databases. Statistical validation demonstrated that structured artistic inputs significantly enriched the yield of valid molecular hits compared with pooled random noise, particularly when using library-based and hybrid generation modes. ImagiChem introduces a new modality for chemical generation that conceptually bridges computer vision, molecular design, and human creativity, thereby expanding the frontier of computational chemistry into the domain of visual cognition.

## Graphical abstract

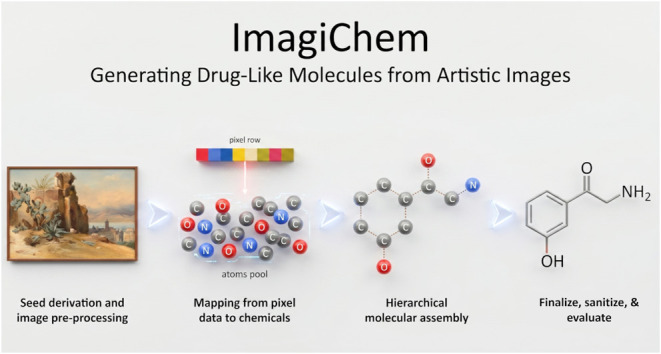

## 1 Introduction

Drug discovery is one of the most complex, time-consuming, and costly endeavors in biomedical research. The development of a single approved drug can take more than 12 years and cost more than 1 billion USD, with only one molecule in 10,000 synthesized reaching the market.^1,2^ Despite remarkable advances in computer-aided drug design (CADD), the discovery process still depends heavily on the incremental optimization of known scaffolds and rational exploration of limited chemical regions.^1–3^ Traditional structure-based CADD (SB-CADD) approaches, such as molecular docking and molecular dynamics simulations, have been accelerated by breakthroughs in structural biology, including the explosion of experimentally determined or artificial intelligence (AI)-predicted protein structures from tools such as AlphaFold.^4^ However, the vastness of the chemical universe, estimated at up to 10^60^ possible small molecules,^1,5^ renders exhaustive searches infeasible, necessitating new paradigms for molecular exploration.

*De novo* drug design (DNDD) has emerged as a transformative strategy for overcoming this challenge. DNDD methods aim to design new compounds from scratch, leveraging heuristic or generative algorithms to traverse the immense chemical space efficiently.^1–3^ The recent rise of deep generative models, including recurrent neural networks (RNNs), variational autoencoders (VAEs), transformers such as MolFormer and ChemBERTa, and diffusion-based molecular generators, has provided powerful means to simulate molecular creativity.^6^ A conceptual comparison between ImagiChem and representative molecular generative paradigms is reported in Table S1, highlighting that ImagiChem is not intended as a distribution-learning competitor to VAE-, transformer-, or diffusion-based models but as a deterministic image-conditioned translational framework.

However, even the most sophisticated architectures remain inherently biased toward known data distributions, as they learn from human-curated chemical data sets (e.g., ChEMBL, ZINC, and PubChem). Consequently, their latent spaces are constrained by precedent, and the probability of generating truly novel chemotypes, remote from training data, is small.^5^

This limitation highlights a paradox in modern drug design: as computational models become more rational and efficient, they may also become less serendipitous. However, serendipity has played a pivotal role in the history of pharmacology. Landmark discoveries, such as penicillin, identified by Alexander Fleming through a chance contamination of a Petri dish,^7^ sildenafil’s unexpected vasodilatory effect (Viagra),^8^ and the analgesic properties of aspirin derived from plant metabolites^9^ all underscore the power of unplanned discovery. True innovation often arises when randomness intersects with structured reasoning,^1,10^ a concept that is typically excluded by computational pipelines.

To reintroduce algorithmic serendipity into molecular generation, we sought an unconventional form of input, rich in structure, yet detached from chemical precedent: visual art. Artworks encode complex patterns of composition, symmetry, color distribution, and form, reflecting a multiscale organization akin to molecular diversity. By translation of these aesthetic features into chemical space via deterministic hierarchical mapping, it is possible to traverse regions of molecular space that conventional models rarely explore.

Herein, we introduce ImagiChem, a generative framework that converts digital artwork into chemically valid molecules via three distinct but complementary generation modes. ImagiChem is fully deterministic; given the same image and generation mode, it consistently produces the same molecular set. Its purpose is not to replicate known QSAR trends or medicinal chemistry heuristics but to act as a translational bridge between aesthetic information and molecular representation. The three modes—Library, From-Scratch, and Hybrid—allow users to navigate a trade-off between curated fragment diversity and unconstrained structural creativity while preserving full reproducibility within each configuration. In doing so, ImagiChem introduces controlled randomness, an “engineered serendipity,” which may lead to unconventional yet valid scaffolds and unexpected chemical architectures.

This paper presents the conceptual and computational foundations of ImagiChem, describes its algorithmic pipeline for converting visual inputs to molecular outputs, and demonstrates its application to a series of historical paintings.

## 2 Results

### 2.1 ImagiChem Pipeline

ImagiChem’s design comprises three generation modes, each sharing the same deterministic seed derivation and image preprocessing foundation (Figure 1). All modes execute four common stages that collectively ensure reproducibility and interpretability:

(i) Deterministic seed derivation and global image profiling.
(ii) Pixel-statistics-to-chemical-alphabet mapping (Library and Hybrid modes).
(iii) Mode-specific molecular assembly.
(iv) Finalization and evaluation.

**Figure 1:**
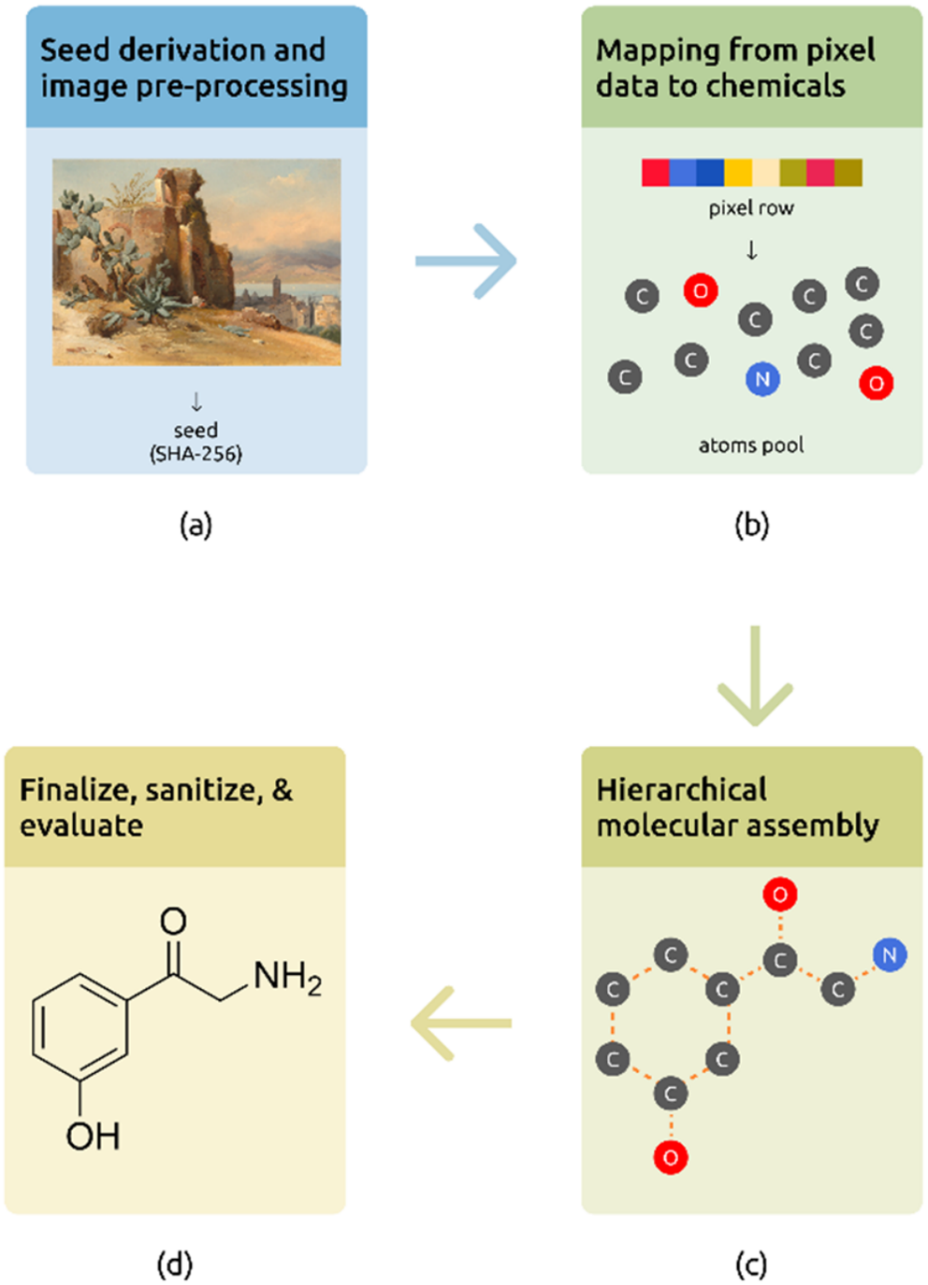
Overview of the ImagiChem pipeline. Stage (a) converts full-color image data into a deterministic seed via SHA-256 hashing and extracts a global artistic profile (spatial coherence, chromatic harmony, and morphological class); (b) encodes per-row pixel statistics, blended with the global profile, into a chemical alphabet; (c) performs mode-specific molecular assembly—Library mode uses the curated Enamine fragment library guided by the morphological class; From-Scratch mode applies a rule-based generator whose blueprint is governed by the global image profile; Hybrid mode combines both; and (d) validates and ranks generated molecules. The image reported in panel (a) is a reproduction of Jean-Charles-Joseph Rémond’s Ancient Ruins near Messina, Sicily (1842), retrieved from the National Gallery of Art Web site, where the media is explicitly indicated as free and in the public domain: https://www.nga.gov/artworks/130895-ancient-ruins-near-messina-sicily.

The three modes are as follows: (1) Library mode, which assembles molecules from the curated Enamine fragment library guided by per-row pixel statistics and a global morphological profile derived from the full image; (2) From-Scratch mode, which generates molecules *de novo* using a rule-based structural blueprint whose ring count, aromatic/aliphatic balance, linker preferences, and decoration patterns are controlled entirely by the global image profile; and (3) Hybrid mode (recommended), which runs both engines independently and merges their unique SMILES outputs into a single ranked list.

### 2.2 Deterministic Seed Derivation and Image Preprocessing

At the core of ImagiChem lies the principle of deterministic reproducibility: given the same input, the system must always yield the same molecular output. Simultaneously, even the slightest visual alteration produced distinct results. This is achieved through cryptographic seed derivation and controlled image preprocessing.

Each run began by converting the input image to grayscale, thereby reducing its dimensionality to a single luminance channel. This analysis focuses on contrast and texture, the structural elements most closely tied to compositional form, while ensuring cross-comparability among artworks with differing color palettes. By operating solely on luminance values, ImagiChem mirrors perceptual mechanisms that emphasize light–dark relationships as the foundation of visual structure.

The full raw byte content of the color image is passed through the SHA-256 cryptographic hash function, which computes a 256-bit digest that serves as the seed for all of the random number generators (RNGs) used throughout the pipeline. By hashing the complete color content rather than a single grayscale row, the seed captures chromatic information (hue and saturation). Owing to the avalanche property of SHA-256, even a single-pixel modification results in a radically different hash, guaranteeing that the corresponding molecular-generation pathway is entirely divergent.

The derived seed initializes all RNGs across Python’s random, NumPy, and custom stochastic libraries, ensuring that every probabilistic decision, from atomic assignment to structural branching, is pseudorandom yet reproducible.^11^ Thus, ImagiChem achieves a balance between sensitivity and determinism: a computational analogue of creative variation, where microscopic changes in visual input cascade into distinct chemical narratives while preserving full reproducibility within each unique artistic configuration.

In addition to the deterministic seed, ImagiChem extracts a global artistic profile from a full-color image. This profile encodes 11 descriptors, including brightness mean and standard deviation, saturation mean and standard deviation, hue standard deviation, edge density, structure strength, spatial coherence, palette harmony, artistic bias, and complexity, and assigns the image to one of five morphological classes (serene_compact, crowded_structured, chromatic_expressive, minimal_ordered, and balanced_painterly). The morphological class modulates core-family selection probabilities, atomic formula targets, and structural blueprint parameters across all generation modes, ensuring that the molecular output is systematically sensitive to the global visual characteristics of the input image.

### 2.3 Pixel Statistics for Chemical Alphabet Mapping

Each row of pixel intensities in an image is treated as a one-dimensional signal, from which ImagiChem extracts descriptive statistics (mean, variance, contrast, and peak count) that are then heuristically mapped to a chemical alphabet. This process, termed molecular dataization, transforms aesthetic or visual signals into structured chemical grammars that are capable of generating plausible molecular compositions.

For each pixel row, ImagiChem extracts a comprehensive suite of local features from both the luminance-weighted grayscale and the per-channel color signals. This feature set included the mean luminance, standard deviation, gradient mean, peak and valley counts, contrast, mean saturation, saturation standard deviation, line coherence, local complexity, and color harmony. To generate a unified representation, these local row-level metrics were integrated with the image’s global artistic profile. This blending utilizes predefined proportional mixing weights to balance local signal characteristics against global aesthetic parameters (such as artistic bias, complexity, spatial coherence, and palette harmony), and the resulting merged feature set holistically reflects both the specific row dynamics and overarching characteristics of the image.

The translation of these engineered features into a molecular composition relies on bounded deterministic mathematical functions:

- Molecular size and carbon framework: The total atom count is computed as a bounded linear combination of local complexity, mean saturation, and artistic bias. This baseline is further adjusted by offsets specific to the image’s assigned morphological class (e.g., adjusting downward for minimal or serene structures and upward for crowded configurations) to maintain the size within a predefined, chemically viable range. The carbon fraction is similarly derived from a bounded function that positively weights artistic bias and coherence, while penalizing excessive saturation.
- Primary heteroatoms (O, N): Oxygen and nitrogen targets are calculated as continuous functions of artistic bias, structural complexity, saturation, and row-level variability. These values are strictly capped by the maximum thresholds tied to specific morphological classes.
- Trace elements (S, halogens): Rare elements are introduced under highly specific and stringent conditions. Sulfur inclusion is limited to a single atom and is permitted only in highly complex, structured, or chromatically expressive regions that surpass strict thresholds of local peak density or spatial coherence. Halogens (restricted to a maximum of one fluorine atom) are incorporated even more sparsely, requiring high spatial coherence, strong artistic bias, specific morphological categorizations, and occurrence strictly at periodic spatial intervals across the pixel rows.

All calculated elemental abundances were truncated to integers, and the total atom count was constrained to strict upper and lower limits. Given a constant initial seed, the entire mapping protocol is deterministic.

To ensure that feature extraction captures genuine structural data rather than signal noise, local peaks were identified by using an adaptive filtering criterion. A pixel is classified as a peak only if its intensity strictly exceeds that of both its immediate neighbors and surpasses a dynamically computed threshold derived from the intensity range of a specific row. This ensures that the algorithm registers only the meaningful local variations.

Ultimately, this algorithmic pipeline yields a linear sequence of atomic symbols (e.g., CCNCO···). This string functions as the foundational elemental input for subsequent processing in both the Library-mode assembly and Hybrid-mode library phase.

In essence, this mapping establishes a consistent and interpretable bridge between *visual structures* and *chemical semantics*, allowing the aesthetic features of an image to emerge as quantifiable molecular blueprints.

### 2.4 Hierarchical Molecular Assembly

Molecular construction within ImagiChem follows a hierarchical, chemistry-inspired assembly process designed to emulate the structured reasoning of synthetic and medicinal chemists, rather than relying on random atomic concatenation. In the Library mode, the system begins by selecting a foundational molecular core from a curated fragment library of 1500 single-pharmacophore fragments (i.e., discrete small molecules each bearing a single binding-capable functional moiety, such as a polar group) from Enamine.^12^ Core selection is not random; each core is preclassified into one of ten structural families (acyclic, simple_carbocycle, simple_heterocycle, aromatic_single, aromatic_fused, mixed_polycyclic, saturated_polycyclic, sulfurized_aliphatic, small_ring, spiro_bridged). Cyclic or polycyclic scaffolds (e.g., benzene, pyrrole, and naphthalene rings) drawn from substructures are commonly found in marketed pharmaceuticals.^13^ The global artistic profile and morphological class define a probability distribution over these families; the target family is selected deterministically by ranking families based on a composite score that combines the family weight, rarity bonus, and antioveruse penalty. Within the chosen family, individual cores are further scored based on morphology-specific bonuses, structural compactness, heteroatom economy, sulfur penalty, and an antireuse term proportional to prior usage in the current run. Such scaffolds serve as a chemically robust foundation for subsequent molecular elaboration, ensuring that the initial hit fragment embodies structural validity, synthetic accessibility (SA), and pharmacological relevance.

The algorithm dynamically selects a core whose atomic requirements can be satisfied by the available atom pool, which is generated from the preceding pixel-to-chemical-mapping stage. Once a suitable core is established, ImagiChem initiates an iterative expansion phase in which functional groups or individual atoms are attached according to property-aware scoring rules guided by the composition of the remaining atom pool, valence constraints, physicochemical targets derived from the artistic profile (target molecular weight, heteroatom count, and ring count), and a novelty penalty that discourages the reuse of previously generated scaffold keys, structural signatures, and functional motifs within the same run. Each addition step respects the chemical bonding rules and explores functional diversity while maintaining overall molecular coherence.

To prevent dead ends in molecular growth, the system continuously monitored the valences of all of the atoms. Suppose that no attachment points remain, but additional atoms are available. In this case, the algorithm may strategically relax the bonding constraints, for example, by converting a double bond into a single bond to open possible sites for substitution. This flexibility allows ImagiChem to explore a wide variety of chemically valid, yet unconventional configurations, thus striking a balance between structural realism and creative diversity.

In effect, this structure-aware compositional hierarchy mirrors the aesthetic logic of artistic layering: stable frameworks (the core scaffolds) provide form and balance, whereas successive modifications introduce complexity and nuance. The result is a computational analogue of compositional harmony, in which molecular architectures emerge through guided hierarchical refinement, faithful to both the principles of synthetic chemistry and the creative dynamics of artistic construction.

### 2.5 From-Scratch Mode

The From-Scratch generator constructs molecules entirely from first principles without using the Enamine fragment library. It operates by first generating a structural blueprint whose parameters—ring count, aromatic/aliphatic ring balance, annulation count, side-chain count, and decoration targets (ethers, methoxy groups, amides, amines, esters, ketones, thioethers, and halogens)—are sampled from distributions conditioned on the global image profile. Ring systems were assembled using a vocabulary of 14 ring types (benzene, pyridine, pyrimidine, pyrazine, imidazole, oxazole, thiophene, cyclohexane, piperidine, morpholine, piperazine, cyclopentane, pyrrolidine, and tetrahydrofuran) connected through six linker modes (direct, methylene, ether, amine, ethyl, and oxyethyl). Decorations are then appended to the available attachment sites. Each candidate was validated against a set of physicochemical constraints (MW, cLog*P*, cLog*S*, TPSA, HBD, HBA, ring counts, sp3 fraction, and rotatable bonds) and scored using a composite metric of ring count, aromatic content, and polar surface area.

### 2.6 Hybrid Mode

In the Hybrid mode, both engines are run independently on the same image using an identical seed and global profile. The Library-based engine is allocated approximately 55% of the progress budget; the From-Scratch engine receives the remaining 45%, using a bitwise-XOR-perturbed seed to maximize structural diversity. The unique SMILES strings from both engines were merged and reranked, yielding a single output list that combined the complementary strengths of curated fragment diversity and unconstrained structural creativity. The Hybrid mode is the recommended default.

### 2.7 Finalization and Validation

The final stage of the ImagiChem pipeline converts internally generated molecular graphs into standardized chemically validated representations suitable for further analysis and application. Each molecular graph was transformed into an RDKit^14^ object, leveraging the library’s robust cheminformatics infrastructure to enforce chemical correctness. Through the Chem.SanitizeMol routine, ImagiChem verifies and corrects the valence states, bond orders, and aromaticity patterns, ensuring that each structure conforms to real-world chemical rules before proceeding to evaluation.

After sanitization, each valid molecule undergoes pharmacological evaluation, which assesses pan-assay interference compounds (PAINS),^15^ that is, compounds that tend to yield false positives during high-throughput virtual screening (HTVS). Furthermore, the synthetic accessibility (SA) of the molecules was quantitatively evaluated by assigning a synthetic difficulty score ranging from 1 to 10.^16^ A score of 1 indicates that the molecules are very easy to synthesize, whereas a score of 10 indicates that the molecules are highly challenging to synthesize. Prioritizing molecules with favorable pharmacological profiles and lower synthetic accessibility scores enables ImagiChem to efficiently filter and classify thousands of generated compounds based on their overall drug-likeness, integrating molecular weight, lipophilicity, hydrogen bonding, polar surface area, and structural simplicity into a single interpretable metric. This automated assessment streamlines the selection process by focusing on candidates with favorable pharmacological profiles, thereby accelerating the downstream experiments.

To further refine the molecular set, duplicate structures were detected and removed via canonical SMILES comparison, ensuring that each retained compound was unique in the output space. The resulting collection of molecules, now fully validated and ranked, is exported as a .CSV file containing the rank, SMILES, drug-likeness score, generation mode, and source image path. Molecules can also be saved as a .mol file or .smi file for downstream computational screening, visualization, or synthesis planning. The graphical interface allows the filtering of results by the SMILES substring and displays the structure of each molecule alongside its drug-likeness score.

In this final stage, ImagiChem unites computational precision with chemical rigor, transforming aesthetically signal-derived abstractions into a curated portfolio of chemically meaningful and pharmaceutically plausible molecular entities.

### 2.8 Application to Artistic Works

To assess the creative and pharmacological potential of ImagiChem, the system was applied to a corpus of ten celebrated artworks (Table 1) spanning diverse periods, styles, and visual structures, which enabled ImagiChem to be tested across a wide range of aesthetic conditions. Renaissance works, such as *The Birth of Venus*, *Mona Lisa*, and *Madonna of the Meadow*, offer balanced compositions and harmonic geometry. Simultaneously, modern pieces such as *Starry Night* and *The Scream* introduce expressive contrasts and high visual entropy. Paintings rich in symbolism and tonal variation, such as *Vucciria* and *Badende Frauen*, further tested the algorithm’s sensitivity to complex visual inputs. Together, this curated selection ensures that ImagiChem’s performance reflects genuine responsiveness to differences in form, color distribution, and luminance, validating its generality across distinct artistic and structural paradigms. All paintings were retrieved from the Artvee Web site (https://artvee.com/) to ensure high-resolution images. Only *Vucciria* and *Virgin Annunciate*, which we wanted to include for personal emotional reasons, were downloaded separately (https://www.theworldofsicily.com/consigli-di-viaggio/12-quadri-famosi-da-vedere-in-sicilia/) because they were not available on Artvee.

**Table 1:** Molecular Diversity Generated by ImagiChem across Ten Artworks (Hybrid Mode)

artwork (artist)	H	W	total molecules	new	existing	NF%
*Bacchus* (Annibale Carracci)	1500	1316	1631	1631	0	100.00
*Bathing Women* (Ernst Ludwig Kirchner)	1452	1800	1482	1482	0	100.00
*Madonna of the Meadow* (Raffaello Sanzio)	4699	3669	4633	4633	0	100.00
*Mona Lisa* (Leonardo da Vinci)	1800	1226	1917	1914	3	99.84
*Starry Night* (Vincent van Gogh)	1391	1800	1753	1739	14	99.20
*The Birth of Venus* (Sandro Botticelli)	1307	2094	1472	1472	0	100.00
*The Scream* (Edvard Munch)	1800	1351	1800	1800	0	100.00
*The Wedding at Cana* (Paolo Veronese)	1718	2528	1821	1821	0	100.00
*Virgin Annunciate* (Antonello da Messina)	675	506	1012	1012	0	100.00
*Vucciria* (Renato Guttuso)	675	692	974	974	0	100.00
total	17,017	16,982	18,495	18,478	17	99.91

Each painting was processed under identical deterministic conditions, enabling a direct comparison of its molecular outputs and the encoded aesthetic–chemical relationships.

Across all images, ImagiChem generated between 675 and 4699 molecules per artwork, depending on the resolution and luminance complexity (Table 1). Each molecule was subsequently cross-referenced with the PubChem database^17^ to determine its novelty. On average, 99.91% of the generated molecules were previously unreported, confirming the system’s ability to consistently explore unexplored regions of the chemical space, regardless of the image dimensions or stylistic origin. This extraordinary rate of molecular innovation highlights ImagiChem’s role as an engine of aesthetic-driven chemical creativity, an algorithmic analogue of artistic inspiration, rendered in molecular form.

### 2.9 Drug-Likeness and Pharmacological Evaluation

To assess the pharmacological plausibility of the molecules generated by ImagiChem, a comprehensive drug-likeness analysis was conducted using three independent predictive models: (1) QED from RDKit, (2) DBPP indices implemented in DBPP-Predictor,^18^ and (3) Drug-likeness and Drugscore indices implemented in DataWarrior.^19^ Each model provides complementary insights into how closely a compound resembles known bioactive molecules in terms of its physicochemical and structural properties.

Prior benchmarking against a reference data set of 2342 FDA-approved drugs (from MedChemExpress) revealed that the DBPP-Predictor offers the most reliable discrimination between drug-like and nondrug-like compounds (Figure 2). Approximately 63% of the approved drugs in this validation set exceeded the DBPP threshold value of 0.736, identified by the model’s authors as the cutoff for favorable drug-likeness.^18^ This result justified the adoption of DBPP as the principal metric for ImagiChem’s pharmacological evaluation.

**Figure 2:**
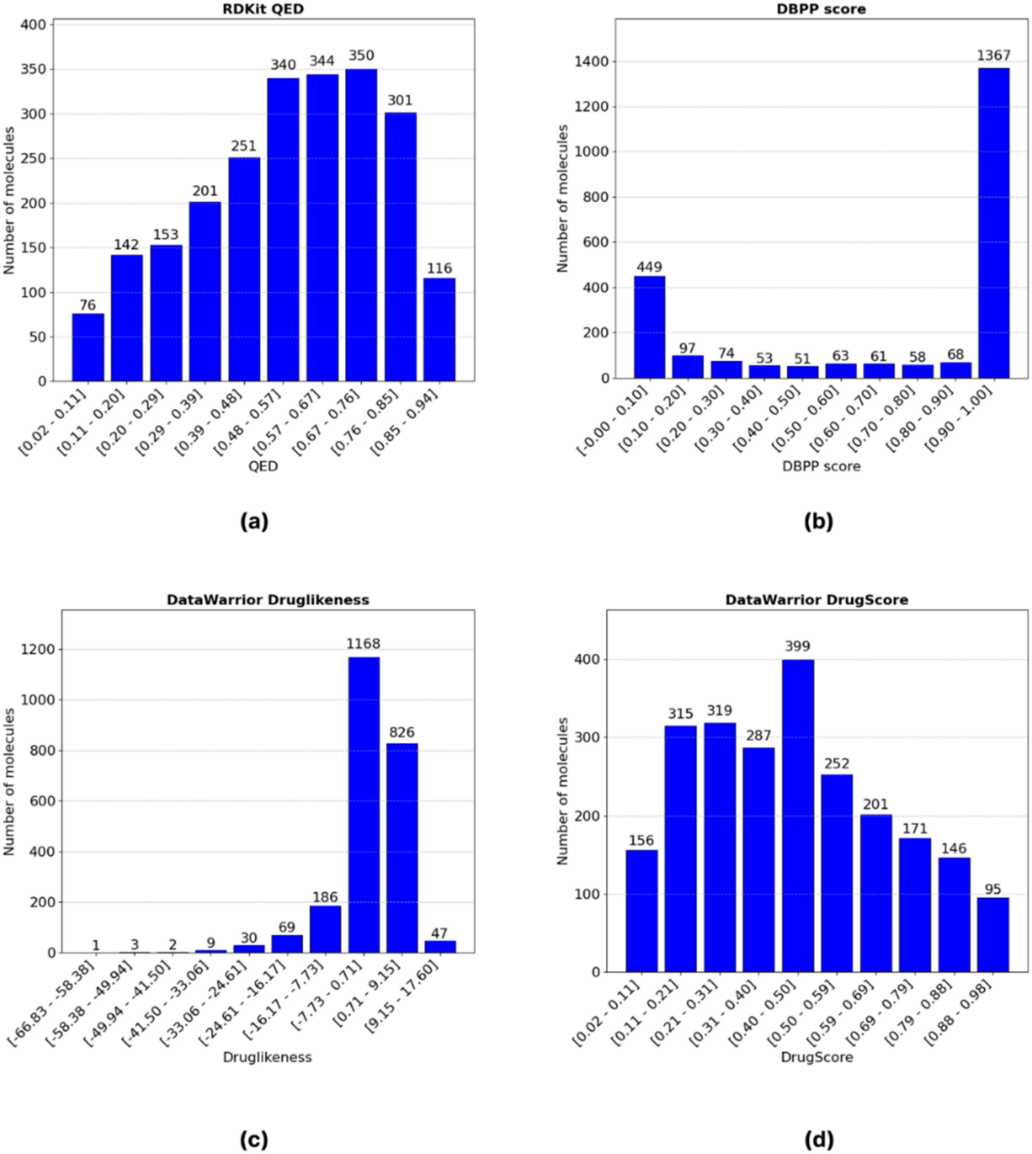
Graphs showing the results of drug-likeness analyses performed by the QED RDKit-based implementation of Biscu-it (a), DBPP scores from DBPP-Predictor (b), Drug-likeness (c), and Drugscore (d) from DataWarrior. For each graph, the *y*-axis shows the number of molecules, and the *x*-axis shows the value ranges, where square brackets indicate that the extreme value is included in the range. Molecules with errors during the QED calculation were not included.

When applied to the complete molecular set generated from the ten analyzed artworks, ImagiChem’s recommended configuration (Hybrid Mode) achieved a mean of 1.31% of molecules exceeding the DBPP threshold (Table 2). Although the absolute proportion of highly drug-like candidates is stringent, this hit rate is highly realistic for *de novo* generation pipelines operating without iterative chemical optimization,^3,5^ representing a highly targeted enrichment of viable chemical spaces. Furthermore, the other generative configurations, Library only and From-Scratch, yielded comparable rates of 1.15 and 2.30%, respectively. The distribution of high-scoring molecules remained heterogeneous across artworks: for instance, *Starry Night* (van Gogh) yielded the highest proportion in Hybrid Mode (3.82%), whereas *The Scream* (Munch) and *The Birth of Venus* (Botticelli) yielded the lowest proportions (0.39 and 0.54%, respectively). This variation reflects the profound influence of the visual entropy and structural composition of each painting on the resulting chemical diversity.

**Table 2:** Drug-Likeness Distribution across Artworks Based on DBPP Threshold (0.736) Calculated in Hybrid Mode (Results about Library Only and From-Scratch Modes Are Available in Tables S2 and S3)

artwork	total molecules	DBPP score >0.736	% above threshold
*Bacchus*	1631	21	1.29
*Bathing Women*	1482	20	1.35
*Madonna of the Meadow*	4633	30	0.65
*Mona Lisa*	1917	35	1.83
*Starry Night*	1753	67	3.82
*The Birth of Venus*	1472	8	0.54
*The Scream*	1800	7	0.39
*The Wedding at Cana*	1821	17	0.93
*Virgin Annunciate*	1012	25	2.47
*Vucciria*	974	13	1.33
total	18,495	243	1.31

A comprehensive overview of the structural diversity, including Bemis–Murcko scaffold uniqueness and mean Tanimoto similarity, alongside the full panel of mean physicochemical and pharmacological metrics (MW, TPSA, QED, SA, Lipinski, Veber, and PAINS) across all generation modes, is provided in Table S4.

High-DBPP compounds predominantly originated from artworks exhibiting specific compositional and textural patterns such as *Starry Night* and *Virgin Annunciate*. Representative examples of these two artworks are shown in Figure 3, illustrating the translation of the visual structures into pharmacologically relevant molecular architectures.

**Figure 3:**
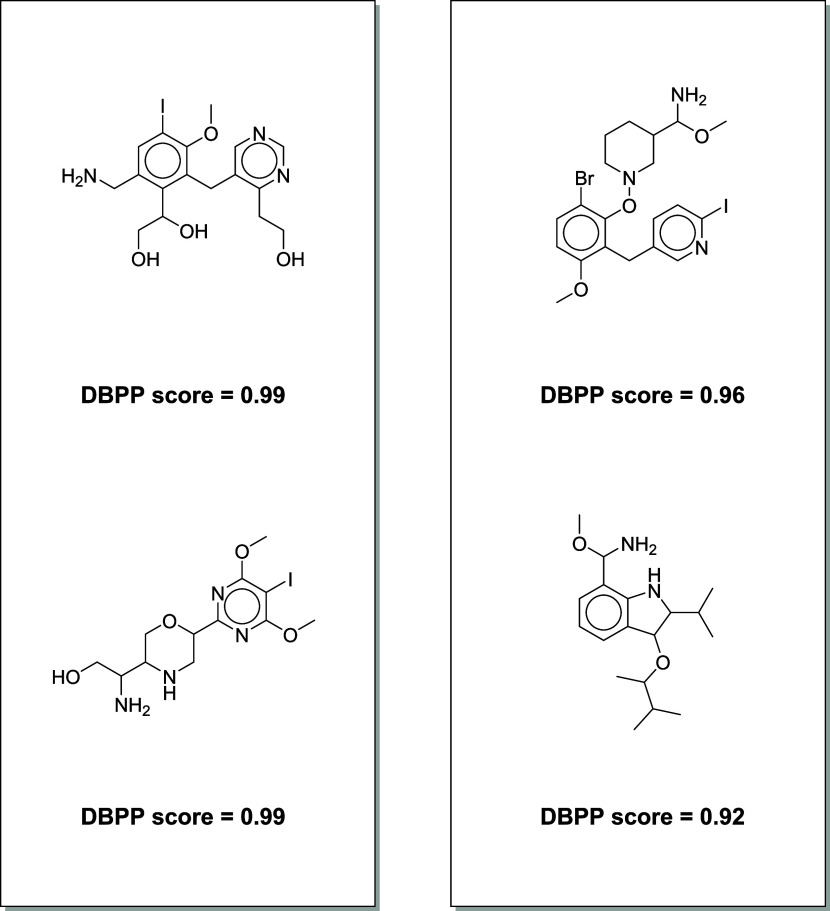
Representative high-DBPP molecules generated by ImagiChem from van Gogh’s *Starry Night* (left panel) and Antonello da Messina’s *Virgin Annunciate* (right panel). The specific aesthetic features of these works correlate with their molecular complexity and aromaticity.

### 2.10 Inverse Virtual Screening (IVS) and Target Prediction

To assess the biological relevance of the aesthetic-driven molecular-generation process, ten representative molecules from the top-ranked DBPP candidates (Figure 4) were subjected to IVS. The analysis followed a previously established validated workflow by our group,^20^ employing the GNINA docking engine to predict ligand–target affinities through convolutional neural network scoring (CNN_VS). Each ligand was screened against a curated panel of 26 experimentally validated protein targets spanning GPCRs, kinases, and enzymes (Table S5), representing a wide range of pharmacological classes.

**Figure 4:**
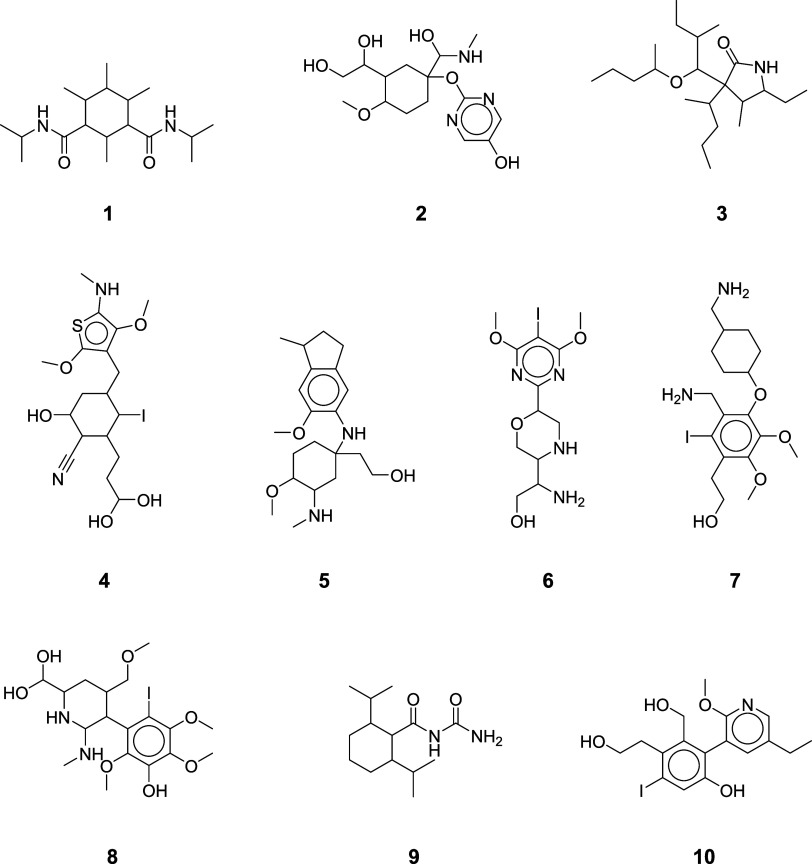
Representative two-dimensional structures of the ImagiChem-generated compounds tested using IVS.

The results, reported in Table 3, demonstrate that several art-derived molecules exhibit predicted binding affinities above the CNN_VS threshold of 6.3, indicating a substantial likelihood of a meaningful ligand–receptor interaction. Notably, compound **3** exhibited a high affinity for the muscarinic M2 receptor, compound **4** displayed predicted activity toward acetylcholinesterase, and compound **7** exhibited multitarget behavior, engaging acetylcholinesterase, epoxide hydrolase, and dopamine D4 receptor.

**Table 3:**
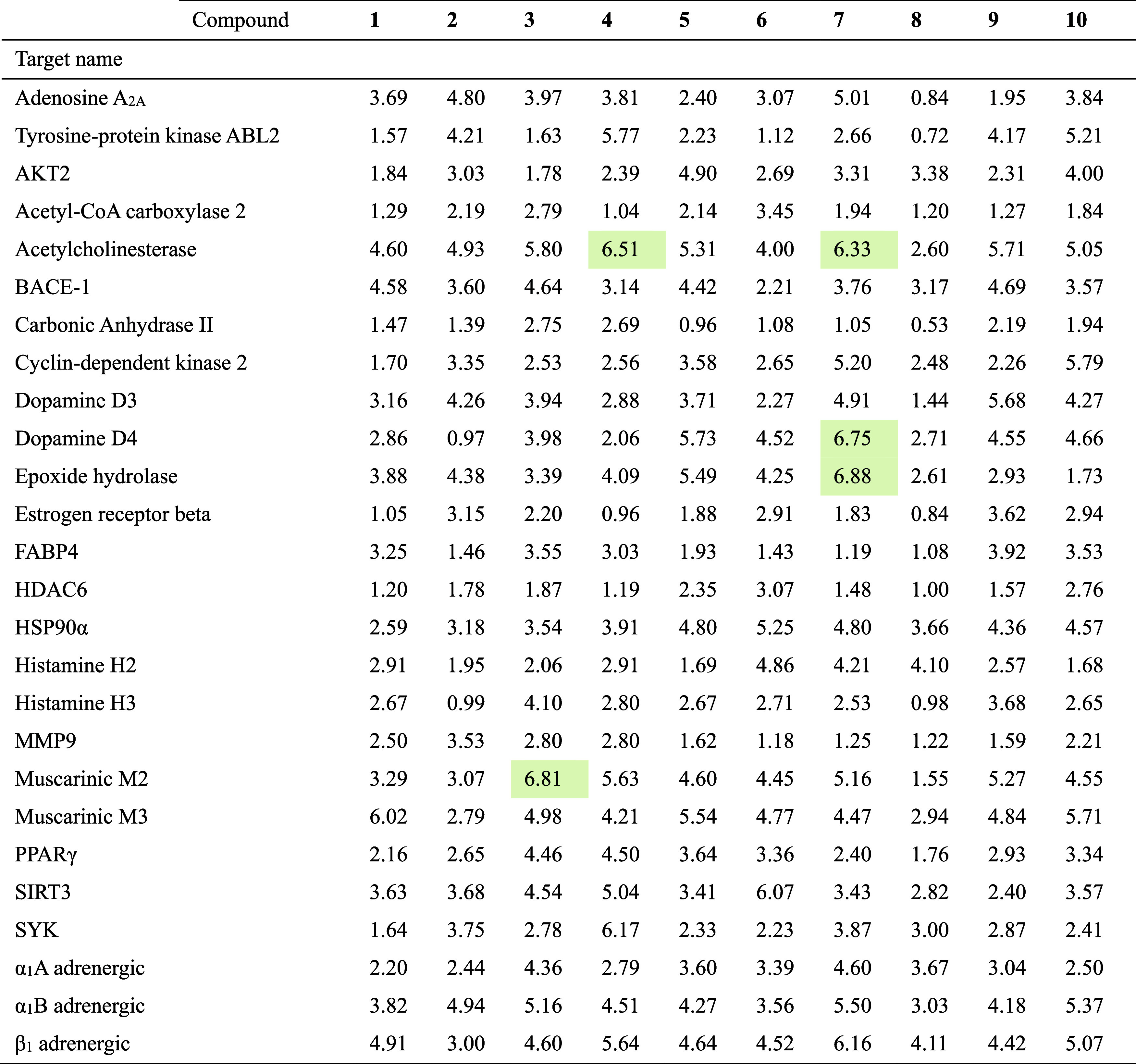
IVS Results for the Representative Ten Compounds with the Highest DBPP Scores Obtained from ImagiChem-Generated Molecules from the Pool of All Paintings^a^

a Each heatmap cell represents predicted ligand–target affinity (CNN_VS value). Molecules exceeding the 6.3 threshold are marked in green, indicating potential pharmacological relevance.

These findings suggest that ImagiChem’s image-guided molecular design can yield scaffolds that are not only chemically valid but also biologically plausible and capable of interacting with key therapeutic targets. The observed polypharmacological tendencies, especially in compound **7**, underscore the algorithm’s potential for generating chemotypes with multitarget profiles, which is a valuable feature in drug discovery for complex diseases.

Further validation through molecular dynamics simulations and experimental assays is necessary to confirm these interactions *in vitro*; however, the present results provide compelling preliminary evidence. Overall, the IVS data reinforce the hypothesis that aesthetic and structural cues extracted from artworks can drive the creation of novel pharmacologically relevant molecular entities, bridging creativity with medicinal chemistry.

### 2.11 Statistical Validation of Generative Methods

We evaluated the performance of the three generative configurations (Hybrid mode, Library only, and From-Scratch) by comparing the molecules derived from paintings with those derived from pooled random noise. The primary performance metric was the absolute hit rate (DBPP score >0.736). The comparative statistical results are summarized in Table 4, and a detailed breakdown of the total molecules generated, absolute hits, and relative hit rates for each mode is provided in Table S6.

**Table 4:** Statistical Validation of Generative Methods through a Comparison between Paintings (P) and Aggregated Random Noise (RN), Evaluated Using Mann–Whitney *U* (MWU), Kolmogorov–Smirnov (KS), and Chi-Square Test (χ^2^) Tests

mode	P hits	RN hits	MWU *p*-value	KS *p*-value	χ^2^ *p*-value
Hybrid mode	243/18,495	17/3217	2.08 × 10^–294^	7.81 × 10^–247^	2.22 × 10^–4^
Library only	195/17,017	4/4000	0	0	1.40 × 10^–9^
From-Scratch	178/7750	34/549	2.94 × 10^–16^	1.12 × 10^–10^	4.99 × 10^–8^

All three methods demonstrated statistically significant divergence between painting- and noise-derived molecules across all evaluated metrics (*p* < 0.001). In the “Library only” and “Hybrid mode” configurations, painting-derived inputs significantly outperformed random noise in generating valid results. Conversely, the “From-Scratch” method exhibited a distinct inversion, with random noise achieving a higher hit rate (6.19%) than paintings (2.30%).

## 3 Discussion

ImagiChem introduces a new computational paradigm, addressing a fundamental limitation of DNDD methods: the inherent bias of in-depth generative models toward known data distributions, which limits their serendipity and the exploration of remote chemical spaces. We reintroduced algorithmic serendipity by using visual art, an input rich in structure but detached from chemical precedents, as a structured prior for molecular generation.

The distinctive strength of ImagiChem lies in its entirely deterministic nature. Unlike stochastic creative AI systems, the algorithm guarantees the full reproducibility of each molecule, which can be traced to a single integer seed cryptographically derived from the input image. This principle is crucial for aligning with the required standards of the scientific rigor.

Applying the pipeline to a corpus of ten artworks demonstrated its ability to generate chemically valid and highly novel molecular sets from diverse visual inputs. Regardless of size or pictorial style, ImagiChem generated molecules with an exceptional novelty rate, averaging 99.91% against the PubChem database, confirming its ability to explore an unexplored chemical space.

Pharmacological evaluation confirmed the biological potential of the generated compounds. Our study identified the DBPP-predictor as the most reliable model for discriminating between drug-like and nondrug-like compounds. Applying this stringent threshold (DBPP > 0.736), we observed rigorous filtering of candidates, with the Hybrid mode yielding 1.31% of molecules within the highly favorable drug-candidate range. While this absolute hit rate is inherently conservative and aligns with realistic expectations for unoptimized *de novo* generation,^3,5^ its true value emerges when compared to that of random noise. As demonstrated in our statistical validation, the Hybrid mode produced 243 valid hits (1.31%) from artistic inputs, compared to only 17 hits (0.52%) derived from pooled random noise. This signifies that the structural and geometric patterns embedded within visual art enrich the yield of viable chemical scaffolds by more than 2-fold compared to random chance. Interestingly, the distribution of molecules with high DBPP scores remained heterogeneous, with works such as *Starry Night* producing the highest proportion (3.82%), demonstrating that specific aesthetic configurations effectively guide the algorithm toward pharmacologically meaningful chemical space.

Even works of contrasting texture and color composition maintained a high level of novelty, reflecting ImagiChem’s robustness across visual domains.

IVS performed on the ten representative top-ranked DBPP candidates provided preliminary evidence of biological relevance. Several candidates showed predicted affinities above the CNN_VS threshold of 6.3 for the key therapeutic targets. Compound **3** showed high affinity for the muscarinic M2 receptor, compound **4** may interact with acetylcholinesterase, while compound **7** has multitarget potential, interacting with the dopamine D4 receptor, acetylcholinesterase, and epoxide hydrolase.

Despite these promising preliminary results, rigorous experimental validation, including molecular dynamics simulations and *in vitro* assays, is essential to confirm the predicted ligand-target interactions.

The very low p-values observed in both the Mann–Whitney *U* and Kolmogorov–Smirnov tests across all three methods demonstrate that the choice of input image (paintings vs random noise) fundamentally alters the algorithmic output. These tests confirm that the generative models are not simply returning random chemical noise; rather, the structural, geometric, and chromatic patterns embedded within human art act as distinct geometric constraints that shift the entire statistical distribution of the generated chemical space. Furthermore, the Chi-Square test validates that the difference in the extremes of these distributions—the “hits” crossing the 0.736 DBPP threshold—is highly robust and reproducible. Beyond these metrics, the results highlight a strong dependency between the type of generative algorithm and the nature of the input data. When the algorithm relies on predefined chemical libraries (“Library only” and “Hybrid mode”), the structured, nonrandom patterns of paintings successfully guide the model toward viable chemical scaffolds, yielding a significant enrichment over noise. Interestingly, the “From-Scratch” mode reveals a profound inversion, wherein pooled random noise outperforms artistic input. We hypothesize that when an algorithm is tasked with building molecular graphs entirely *de novo* without library constraints, the highly ordered patterns of paintings may act as overly rigid boundaries, effectively trapping the generator in suboptimal, local minima. In contrast, the high entropy of random noise provides the necessary stochastic flexibility, allowing the “From-Scratch” algorithm to explore a wider, unconstrained chemical space to find valid hits. Ultimately, while the “Library only” approach demonstrated the highest relative enrichment of paintings over noise (11.5×), we strongly recommend the Hybrid mode for practical applications in drug discovery and molecular design. In real-world pharmaceutical research, the primary objective is not merely to maximize the statistical contrast against a noise baseline but to maximize the absolute yield of structurally novel, high-quality molecular hits. From this pragmatic perspective, the Hybrid mode emerges as the superior architecture, generating the highest absolute number of painting-derived hits compared to the “Library only” approach.

Looking to the future, ImagiChem provides a conceptual foundation for evolution toward multimodal generative architectures. The current three-mode design—Library, From-Scratch, and Hybrid—demonstrates that structurally distinct generation philosophies can be systematically conditioned on visual inputs and combined to maximize chemical diversity. Future improvements will focus on integrating joint image–molecule embeddings (e.g., via CLIP-type encoders and Transformer-based decoders), implementing explainable attention mechanisms that directly link global image features and morphological classes to specific molecular substructures, and using reinforcement learning to further optimize scaffolds for drug targets while preserving their aesthetic origins.

In conclusion, ImagiChem demonstrates that nonscientific human artifacts and works of art can serve as a creative and structured complement to rational drug design. The combination of high molecular novelty, the rediscovery of existing drugs, and the identification of pharmacologically plausible candidates marks the inauguration of a new field: aesthetic chemistry, in which the compositional harmony of art algorithmically merges with molecular functionality.

## 4 Methods

### 4.1 Overview of the Computational Workflow

The software takes a color image as input and supports three generation modes (Library, From-Scratch, and Hybrid), which can be selected from the graphical interface. In the Library mode, each row of pixels produces an “extended” string of atomic symbols that are assembled into a molecular graph using a curated library of cores and functional groups. In the From-Scratch mode, the global image profile drives a rule-based structural generator. In the Hybrid mode, both engines run in sequence, and their outputs are merged. In all modes, the molecules were converted to RDKit Mol objects, sanitized, and scored.

The main computational steps are as follows:

1. *Seed Generation*. A deterministic random seed is derived from the SHA-256 hash of the full-color image’s byte content (image_to_seed). Hashing the color content, rather than a single grayscale row, preserves the chromatic information used by the global artistic profiles. This ensures the complete reproducibility of all random operations (random.seed, numpy.random.seed, and RNG.seed).
2. *Image Processing*. The image was read in grayscale and split into rows of pixels (split_pixel_rows).
3. *Row-Level Molecular Encoding*. For each row:
  - Row statistics were computed (PixelLineAnalyzer.analyze_pixel_pattern).
  - These statistics were mapped to atomic counts (PixelLineAnalyzer.generate_molecular_formula_string).
  - A molecular assembly is generated from the resulting string (assemble_from_input_string).
  - The structure was converted to an RDKit molecule and sanitized, and 2D coordinates were computed.
4. *Postprocessing*. The generated SMILES strings were refined (refine_smiles), and the SA score was computed (calculate_sa_score, rescaled to 0–10). Pan-assay interference compound (PAINS) filters were also applied for drug-like evaluation.
5. *Output*. The molecules are ranked by increasing SA, and a list of (SMILES, SA score, PAINS filters) tuples is generated.

### 4.2 Deterministic Seeding and Random Noise Controls

To guarantee the reproducibility of all stochastic molecular-generation steps, the system relies on a master seed derived directly from the input image. Specifically, the raw byte content of the color image is processed through a SHA-256 hash function to produce an integer seed. This approach preserves essential chromatic data, aiding in the algorithmic differentiation between structured artistic input and pure random noise. This master seed initializes all internal pseudorandom number generators. Furthermore, decision-making nodes within the graph assembly process—such as the selection of topological cores, functional groups, and discrete atom additions—utilize a custom deterministic unit function. This function hashes dynamically constructed string signatures, which incorporate contextual states like profile metrics and remaining atom pools, to produce consistent probabilistic decisions between 0.0 and 1.0. In addition to absolute determinism, the pipeline incorporates explicit mathematical controls to quantify and mitigate visual noise. To establish baseline statistics that are robust against microscopic variations, grayscale matrices are initially downsampled to a 256 × 256 resolution using area interpolation. A Gaussian blur (σ_*x*_ = 3.0) is subsequently applied, and the absolute pixel-wise difference between the downsampled and blurred matrices is calculated to extract a quantitative fine noise metric. This quantified noise inversely modulates the spatial coherence parameter; consequently, higher noise levels strictly penalize spatial coherence. This noise-adjusted coherence directly influences downstream heuristic weights, altering the target number of aromatic rings, drug-likeness optimizations, and the probability distributions of specific morphological scaffold families. Finally, local one-dimensional pixel rows are continuously evaluated for flat gradients to bypass peak-finding logic errors on uniform or zero-noise lines, ensuring that the system adapts its chemical output strictly to the structural integrity of the input.

### 4.3 Mapping from Pixel Statistics to Atomic Counts

All numerical mappings are implemented in PixelLineAnalyzer.generate_molecular_formula_string, which fuses per-row pixel statistics with the global artistic profile via blend_line_with_global_profile.

#### 4.3.1 Profile Blending

Per-row local features (mean luminance, standard deviation, gradient mean, peak count, valley count, contrast, saturation mean and standard deviation, line coherence, local complexity, and color harmony) are blended with the global artistic profile features using fixed mixing weights as follows:

- artistic_bias = 0.6 × global_artistic_bias + 0.4 × line_coherence;
- spatial_coherence = 0.7 × global_spatial_coherence + 0.3 × line_coherence;
- complexity = 0.6 × global_complexity + 0.4 × local_complexity;
- palette_harmony = 0.7 × global_palette_harmony + 0.3 × color_harmony.

This produces a merged feature set that reflects both the row-level signals and global characteristics of the image.

#### 4.3.2 Total Atom Count and Carbon Fraction

The approximate total atom count was derived as

- total_atoms = clip­(20 + 18 × complexity + 6 × saturation_mean – 8 × artistic_bias – morphology_offset, 18, 42)

where morphology_offset is −3.5 for serene_compact, +2.0 for crowded_structured, −2.0 for minimal_ordered, and 0 for the remaining classes.The carbon fraction is:

- carbon_fraction = clip­(0.58 + 0.16 × artistic_bias + 0.06 × spatial_coherence – 0.05 × saturation_mean, 0.56, 0.82)

yielding a carbon count of c_count = clip­(round­(total_atoms × carbon_fraction), 14, 34).

#### 4.3.3 Element-Specific Rules

Oxygen: o_target = clip­(round­(1 + 1.1 × (1 – artistic_bias) + 0.5 × complexity + 0.7 × saturation_mean), 1, 4).

Nitrogen: n_target = clip­(round­(1 + 0.9 × (1 – artistic_bias) + 0.4 × complexity + 0.4 × std/90), 1, 4).

Sulfur: count = 1 only when the morphological class is crowded_structured (with complexity > 0.76, spatial_coherence > 0.52, and local peak count above row_length/18) or chromatic_expressive (with complexity > 0.80 and artistic_bias < 0.46); otherwise, count = 0.

Halogens (F, Cl, Br, I): at most one fluorine atom is added, and only when artistic_bias > 0.62, spatial_coherence > 0.58, morphological class is serene_compact or balanced_painterly, and the row index satisfies i % 37 = = 0.

All per-element counts were clipped such that the total atom count remained in the range of [18, 46]. If the cumulative heteroatom budget is exceeded, then the oxygen and nitrogen targets are scaled down proportionally, and sulfur is dropped unless the morphological class permits it and the budget accommodates at least five heteroatoms.

#### 4.3.4 Final Atomic Sequence

The atomic symbols were concatenated according to their counts ([atom] × count), shuffled via rng.shuffle (formula_parts), and joined into a single continuous string (e.g., “CCNCO···”).

#### 4.3.5 Rounding and Fraction Handling

All floating-point results were truncated to integers using int­(), corresponding to truncation toward zero. No stochastic rounding was applied. The mapping process is entirely deterministic, given a fixed seed and image.

### 4.4 Row Aggregation Strategy

The rows were processed independently.

Each pixel row yields its own atomic and molecular assemblies. No global atom pools are accumulated across the rows. This design is implemented in run_imagichem_processing, which iterates over the rows, constructs each molecular formula, and processes them separately.

### 4.5 Curated Libraries of Cores and Synthons

The provided groups.py file defines a minimal example library (GROUP_LIBRARY) containing six functional groups (ester, amide, amine, alcohol, nitro, and ether). Each entry includes:

- a factory function generating a molecule graph fragment;
- atomic requirements;
- probabilistic weights.

The cores.py file provides the foundational molecular cores of a curated fragment library containing 1500 single-pharmacophore fragments from Enamine. Each core is automatically characterized at the start-up by its topological family (one of ten classes: acyclic, simple_carbocycle, simple_heterocycle, aromatic_single, aromatic_fused, mixed_polycyclic, saturated_polycyclic, sulfurized_aliphatic, small_ring, spiro_bridged), ring sizes, aromatic fraction, heteroatom count, and spiro/bridgehead status. These metadata are used at runtime to select cores whose families match the morphological class of an input image.

The use of fragments bearing a single pharmacophore (rather than multiple, distally separated functional groups) is advantageous: this simplifies the binding interaction to one key moiety, allowing (i) higher synthetic tractability of fragment growth (fewer conflicting vectors and functional groups to optimize) and (ii) more straightforward interpretation of structure–activity relationships and binding mode evolution.

From a fragment-based drug discovery (FBDD) perspective, designing a library around such simplified, single-pharmacophore entities is aligned with best practice: fragments are, by definition, low in molecular weight and complexity (following, e.g., the “Rule of Three”: MW <300, ≤3 H-bond donors, ≤3 H-bond acceptors, cLog*P* ≤3) and yet high in atom-efficiency, thereby enabling a small library to sample chemical space efficiently.

The Enamine library further ensures that all compounds pass strict “Ro2-like” physicochemical and structural filters (excluding PAINS, reactive, and toxic motifs), making initial hits less likely to be false positives and synthetically tractable for follow-up studies.

By anchoring such well-defined, stable fragments and scaffolds, the workflow facilitates a “hit to lead” transition in which the fragment core is elaborated (via fragment growth, merging, linker insertion, or scaffold extension) in a controlled manner, preserving the original binding moiety while adding vectors for potency, selectivity, and ADMET optimization. The literature shows that high-quality fragment libraries (with careful attention to diversity, pharmacophore novelty, and synthetic follow-up potential) are critical for the success of FBDD campaigns, especially for challenging targets.^21^

In short, the fragment library provides a chemically validated seed (a fragment with a known drug-like core and a single binding pharmacophore), the scaffold provides the structural backbone, and from there, a medicinal chemistry trajectory can be laid out in a modular, rational fashion.

### 4.6 From-Scratch Generator Parameters

The rule-based From-Scratch generator constructs molecules entirely from first principles, without drawing on the Enamine fragment library. It operates by first generating a structural blueprint, whose parameters are sampled from distributions conditioned on the global image profile. The blueprint specifies the following:

- ring_count ∈ {2, 3, 4} (sampled with weights 0.15/0.55/0.30);
- aromatic_rings ≤ ring_count (weights 0.15/0.55/0.30);
- annulations ∈ {0, 1, 2} (weights 0.18/0.55/0.27);
- linked_ring_extensions ∈ {1, 2, 3} (weights: 0.18/0.58/0.24);
- side_chains ∈ [2, 4];

and decoration targets for amides (0 in 92% of cases), amines (0/1/2), esters (0/1), ketones (0/1), ethers (1/2/3), methoxy groups (0/1/2), hydroxyethyl groups (0/1), alcohols (0/1/2), nitriles (0/1), thioethers (0/1), and halogens (0/1/2).

Ring systems are assembled from a vocabulary of 14 ring types (benzene, pyridine, pyrimidine, pyrazine, imidazole, oxazole, thiophene (aromatic), cyclohexane, piperidine, morpholine, piperazine, cyclopentane, pyrrolidine, and tetrahydrofuran (aliphatic)) using weighted selection from the “balanced” preset. Connections between rings use one of six linker modes: direct (weight = 0.28), methylene (0.24), ether (0.16), ethyl (0.14), oxyethyl (0.10), and amine (0.08). Once the ring scaffold is established, decorations are appended to the available attachment sites in the following order: ethers, methoxy groups, hydroxyethyl groups, alcohols, amines, amides, esters, ketones, nitriles, thioethers, halogens, tertiary-amine-like groups, and saturated side chains.

Each candidate molecule was validated against the BALANCED_CONSTRAINTS set: MW ∈ [150, 600] Da, cLog*P* ∈ [−5, 5], TPSA ∈ [0, 335] Å^2^, HBD ∈ [0, 5], HBA ∈ [0, 10], rings ∈ [1, 4], sp3 fraction ∈ [0.18, 0.82], and rotatable bonds ≤ 8. Invalid candidates (those violating any constraint, containing carbamate groups, or having more than one spiro atom) were discarded. Accepted candidates are scored by a 16-component composite that rewards QED, ring count (optimum 2–3), aromatic ring count (optimum 1–2), cLog*P* ∈ [−0.5, 4.0], TPSA ∈ [30, 115] Å^2^, HBD ∈ [0, 2.5], and HBA ∈ [1, 7], and penalizes excessive amide, carbamate, and ester content, large numbers of rotatable bonds, and extreme sp3 fraction values.

In the Hybrid mode, the From-Scratch engine is seeded with image_seed XOR 0 × 5A5A1357 to maximize structural diversity relative to the library engine; the target molecule count is set to max­(120, min­(target_*n*/2, 1200)), where target_*n* = max­(60, min­(number_of_pixel rows, 800)).

### 4.7 Parameters of the Hierarchical Assembly

#### 4.7.1 Core Selection and Insertion

Core selection follows a two-stage, morphology-informed process. First, structural family weights are derived from the morphological class; available families are ranked by a composite score combining the family weight, rarity bonus (1/√count), and antioveruse penalty based on observed vs target family share. The top-scoring family was deterministically selected. Second, individual cores within the chosen family are scored by morphology-specific bonuses, structural compactness (5–11 atoms, 1–2 rings preferred), heteroatom economy, sulfur penalty, and an antireuse penalty proportional to the prior usage in the current run.

- Number of Cores (*n*_cores).

Defaults to 1; *n*_cores = 2 only when sum­(pool.values­()) ≥ 24, morphological class is crowded_structured, complexity > 0.68, artistic_bias < 0.48, and spatial_coherence < 0.48.

#### 4.7.2 Main Assembly Loop

The main loop runs while sum­(pool.values) > 0.

Each iteration:

- Identifies eligible functional groups whose atomic requirements are satisfied.
- Each group has an independent insertion probability given by its weight (e.g., 0.45 for esters and amides).
- If a group is selected, an attachment site (host) is chosen using choose_host­(g), and the group is merged into the graph. The atomic requirements were then subtracted from the pool.

If no group is selected, a fallback rule adds a single atom chosen by choose_next_elem_for_chain­(pool), which determines the element with the highest valence that is still available.

#### 4.7.3 Host Selection

Hosts are chosen from atoms with remaining valence (valence_rem ≥ 1), sorted by (number of neighbors, – remaining_valence) to prefer less-substituted sites.

A random selection is made among the top 50% (up to 6 candidates).

#### 4.7.4 Double-Bond Demotion and Valence Relaxation

The function demote_some_double_bonds_until_capacity­(g, needed_hosts) converts double bonds (preferentially involving carbon) to single bonds until enough attachment sites are available.

This function is invoked when the number of available hosts is insufficient for the addition of new fragments.

#### 4.7.5 Final Connectivity

Disconnected components (components­(g)) are connected iteratively.

Up to 200 attempts are made to join small components through the available host atoms. Each successful connection consumes one unit of valence from each atom involved.

### 4.8 RDKit Conversion and Sanitization

#### 4.8.1 Conversion

graph_to_rwmol­(g) builds a mutable RDKit molecule (Chem.RWMol) by Adding Atoms and Bonds (BondType.SINGLE, DOUBLE, and TRIPLE).

#### 4.8.2 Sanitization

- Default RDKit Sanitization

Chem.SanitizeMol­(mol) is called with default flags.

- Fallback.

If sanitization fails, all double bonds are demoted to single bonds (g.demote_bond) and the molecule is rebuilt and sanitized.

In the present study, stereochemical configurations were not assigned to the compounds that were generated. This choice was deliberate and reflected both the methodological and conceptual considerations. The primary objective of the generation process was to explore topological and pharmacophoric diversity rather than to model specific three-dimensional conformations. Because stereochemistry does not alter the two-dimensional connectivity of atoms but merely defines their spatial arrangement, omitting stereochemical information allows the system to operate within a simplified yet chemically meaningful representation of molecular space.

Furthermore, the construction procedure relies on fragment-based assembly and topological rules without explicitly evaluating three-dimensional energies or conformational optimizations. Under such conditions, any stereochemical assignment, whether random or deterministic, would be arbitrary and unsupported by an underlying physical model. Including stereocenters without energetic or conformational validation could lead to ambiguous and chemically inconsistent results.

Finally, by excluding stereochemical specifications, the generated SMILES remain canonical and unambiguous, facilitating downstream processing, comparison, and validation of molecular structures. Stereochemistry can be introduced at a later stage, if required, through dedicated conformational analysis or stereoselective enumeration once energetically and pharmacologically relevant configurations have been meaningfully defined.

2D coordinates were generated by using AllChem.Compute2DCoords­(mol) for visualization purposes only.

### 4.9 Probabilistic and Numerical Parameters

The deterministic nature of ImagiChem depends on a defined set of numerical mappings, probability weights, and structural thresholds that govern the translation of image-derived descriptors to molecular-generation rules. For transparency and reproducibility, the principal parameters controlling atom count assignment, functional group insertion, core selection, and assembly attempts are summarized in Table 5.

**Table 5:** Principal Probabilistic and Numerical Parameters Used in the Deterministic Image-to-Molecule Conversion Workflow

parameter	description	value/range
image seed	SHA-256 hash of image bytes	deterministic
mapping mean → C	[0, 255] → [15, 40]	linear
mapping contrast → total atoms	[0, 255] → [10, 80]	linear
mapping mean → O	[0, 255] → [1, 10]	linear
mapping std → N	[0, 100] → [1, 8]	linear
peak threshold	_find_peaks/_find_valleys	0.3
S presence rule	num_peaks >250	binary
halogens	allowed every 20th row	0–1
group insertion probability	weight in GROUP_LIBRARY	0.15–0.55
max connection attempts	200	
number of molecular cores	1 or 2	depends on the atom pool size

### 4.10 RDKit and Library Versions

The program requires RDKit (Chem, AllChem, SA_Score, Descriptors, and Draw) and auxiliary packages (NumPy, OpenCV, and PubChemPy).

For reproducibility, the specific version number of each is stated (RDKit 2025.9.1, NumPy 2.3.3, OpenCV 4.12.0.88, Pillow 10.2.0, and PyQt6 6.9.1).

### 4.11 RDKit Sanitization

The molecules were sanitized using Chem.SanitizeMol in RDKit. The SanitizeMol function in RDKit attempts to validate and standardize chemical structures. This includes critical tasks, such as (i) determining aromaticity, (ii) calculating hybridization, and (iii) validating valence. If the SanitizeMol function fails, then a recovery fallback is initiated to resolve the valence issues and allow the crucial RDKit molecule sanitization step to be retried.

### 4.12 Molecular Docking

Protein structures were prepared using YASARA (version 25.1.13)^22^ at a physiological pH (7.4). The 3D structures of the ligands were generated and minimized using Open Babel (v. 3.1.1),^23^ reflecting the physiological pH (7.4) state. Docking simulations were performed using GNINA (v. 1.3)^24^ in rescoring mode with CNN_VS scoring functions. Binding pockets were defined from cocrystallized ligands using AutoDock Tools (v. 1.5.7).^25^

### 4.13 PubChem Similarity Search Script

Searching PubChem is handled by the pubchem_check.py script, which implements a cheminformatics pipeline for the batch structural screening of compounds against the PubChem (NLM) database. The system was configured to perform only an exact structural matching. The core logic is encapsulated in the get_pubchem_results­() function, which uses the pubchempy library to search for a direct match to the provided SMILES. For each SMILES, the script returns the associated CID (compound ID) if found in PubChem or the NEW flag if the compound is not present in PubChem. The results were processed in batches from the input.*csv* file and written to a Separate tab-delimited *.txt* file.

### 4.14 QED and DBPP Calculations

The QED and DBPP scores were computed using internal scripts provided in the GitHub shared repository and the DBPP-Predictor GUI, respectively, with the default settings. Similarity checks against PubChem were performed by using PubChemPy.

### 4.15 Generation of Random Noise Control Images

To establish a statistically rigorous negative control baseline, uniform white noise images were procedurally generated using a custom Python script relying on the NumPy library. The control data set consisted of synthetic RGB images with a resolution of 1000 × 1000 pixels. To thoroughly evaluate the algorithm’s sensitivity to chromatic complexity, the noise was generated at four distinct color depths: 3-bit, 6-bit, 12-bit, and 24-bit RGB.

For each bit depth configuration, pixel intensity values for the Red, Green, and Blue channels were sampled independently from a discrete uniform distribution. Specifically, the number of available intensity levels per channel was defined as 2^(*n*/3)^, where *n* is the total bit depth. The sampled integer values were subsequently linearly scaled to the standard 8-bit per channel range (0–255) to construct valid image arrays. This procedural approach guarantees that the negative control strictly lacks any spatial coherence, structural gradients, or aesthetic composition, thereby providing a purely entropic baseline of visual data.

### 4.16 Statistical Validation of Generative Methods

Statistical evaluations were performed using Python (via the Pandas, NumPy, and SciPy.stats libraries) to assess whether the image source (paintings vs noise) significantly influenced the quality of the generated molecules. Three distinct statistical tests were employed, each providing a specific interpretive lens: (a) Mann–Whitney *U* test (two-sided) is a nonparametric test used to evaluate whether the global medians and bulk distributions of DBPP scores differed significantly between the painting-derived and noise-derived cohorts; (b) Kolmogorov–Smirnov (KS) test compares the empirical cumulative distribution functions of the two sets; it is highly sensitive to differences in the shapes and tails of the score distributions, allowing us to confirm if the models explored fundamentally different chemical spaces; and (c) Chi-Square test of Independence evaluates the practical success of the models, and we calculated the absolute hit rate (percentage of molecules crossing the >0.736 DBPP threshold). A contingency table (Hits vs Nonhits for both data sets) was constructed, and the Chi-Square test was applied to determine if the enrichment of high-scoring molecules was statistically significant rather than due to random chance.

## Supplementary Material



## Data Availability

All data supporting this study are publicly accessible on Zenodo (https://zenodo.org/records/17549410). The ImagiChem code and the generated molecules are available on GitHub (https://github.com/rocco-b/ImagiChem). Furthermore, to ensure full reproducibility and platform independence, given that minor variations can arise from differences in library or interpreter versions, we compiled the entire codebase into standalone executables for both Windows and Linux (ImagiChem-1.0-win64.msi and ImagiChem-1.0-linux, respectively), which are also available in the project repository.

## Notes

Conceptual comparison between ImagiChem and representative molecular generative paradigms (Table S1); drug-likeness distribution across artworks based on DBPP threshold (0.736) calculated on the From-Scratch mode (Table S2); drug-likeness distribution across artworks based on DBPP threshold (0.736) calculated on the Library Only mode (Table S3); structural diversity and physicochemical profiles of ImagiChem-generated molecules across modes (Table S4); the protein models utilized during the IVS (protein) phase (Table S5); a comparative performance summary: artistic paintings vs random noise across generation modes (Table S6) (PDF)

## Notes

In memory of Professor Carmela Spatafora, a dear friend, colleague, and valued scientist, on the tenth anniversary of her untimely passing.
