# Supplementary material for: ImagiChem: Hybrid Deterministic Image-Conditioned Generation of Chemically Valid and Drug-like Molecules from Artistic Inputs
Source: ACS Omega. 2026 Jul 9;11(28):42923–36. doi: 10.1021/acsomega.6c05429 (PMC13393192; doi:10.1021/acsomega.6c05429)
Supplement: Supplementary file 1 [file ao6c05429_si_001.pdf]

Supporting Information

ImagiChem: Hybrid Deterministic Image-Conditioned Generation of Chemically Valid and Drug-like Molecules from Artistic Inputs

Rocco Buccheri and Antonio Rescifina*

Department of Drug and Health Sciences, University of Catania, Viale A. Doria 6, 95125 Catania, Italy

TABLE OF CONTENTS

Table S1. **Conceptual comparison between ImagiChem and Representative Molecular Generative Paradigms.[a]**

Table S2. **Drug-Likeness Distribution Across Artworks Based on DBPP Threshold (0.736) calculated on From Scratch mode.**

Table S3. **Drug-Likeness Distribution Across Artworks Based on DBPP Threshold (0.736) calculated on Library Only mode.**

Table S4. **Structural diversity and physicochemical profiles of ImagiChem-generated molecules across modes. Data includes total valid molecules, unique Bemis-Murcko scaffolds (Unique BM), and scaffold diversity (%). Internal structural diversity is calculated as 1 minus the mean Tanimoto similarity (Morgan fingerprints, radius = 2). Pharmacological evaluation reports mean Molecular Weight (MW), Topological Polar Surface Area (TPSA), Quantitative Estimate of Drug-likeness (QED), and Synthetic Accessibility (SA) scores, alongside the percentage of molecules passing Lipinski's Rule of Five, Veber's rules, and triggering PAINS alerts.**

Table S5. **This Table Summarizes the Protein Models Utilized During the IVS (Protein) Phase. For Each Model, the Identification Code (PDB ID) is Provided, Along with the Source Database from which it was Obtained: the Protein Data Bank (PDB) or PDB-REDO (REDO). Additionally, the Spatial Coordinates Defining the Center of the Docking Simulation Box (Center x, Center y, Center z) are Listed, as are the Dimensions of the Simulation Box Along Each Axis (Npts x, Npts y, Npts z). A Uniform Spacing Value of 1 Å was Applied Across All Simulation Boxes to Maintain Consistency in Grid Resolution**

Table S6. **Comparative Performance Summary: Artistic Paintings *vs.* Random Noise across Generation Modes.**

Table S1. Conceptual comparison between ImagiChem and Representative Molecular Generative Paradigms.[a]

Generative paradigm	Typical input / representation	Training required	Primary molecular prior	Deterministic output for identical input	Image-conditioned by design	Main purpose	Typical validation	Relationship to ImagiChem
VAE-based molecular generators	SMILES strings, molecular graphs, or latent molecular vectors	Yes	Learned latent distribution from molecular datasets such as ZINC, ChEMBL, or related collections	No, generally stochastic unless sampling is fixed	No	Learn a continuous latent space for interpolation, reconstruction, optimization, and property-driven generation	Reconstruction, validity, novelty, uniqueness, property optimization, latent-space smoothness	Data-driven distribution-learning paradigm; useful benchmark conceptually, but not directly equivalent because ImagiChem does not learn from molecular datasets
Junction-tree/graph VAE generators	Molecular graphs decomposed into substructure trees and graph assemblies	Yes	Learned graph/substructure statistics from known molecules	No, generally stochastic unless sampling is fixed	No	Generate chemically valid molecular graphs through learned substructure assembly	Validity, novelty, uniqueness, property optimization, graph validity	Shares the idea of chemically constrained assembly, but differs because ImagiChem uses deterministic image-derived rules rather than learned graph priors
Transformer / MolGPT-like generators	Autoregressive SMILES sequence generation	Yes	Learned token-level chemical syntax and molecular distribution from training sets	No, generally stochastic unless decoding is fixed	No	Generate new SMILES by next-token prediction, optionally property- or scaffold-conditioned	Validity, uniqueness, novelty, property optimization, benchmark suites such as MOSES/GuacaMol	Represents state-of-the-art sequence generation; ImagiChem differs because visual input, not a learned molecular language model, drives molecular construction
Diffusion-based molecular generators	Molecular graphs, 3D coordinates, conformers, or molecular fields	Yes	Learned denoising trajectory from molecular data distributions	No, generally stochastic unless sampling path and noise are fixed	No, unless specifically extended to image-conditioned molecular design	Generate molecules or conformers by iterative denoising, often with geometric or property constraints	Validity, stability, 3D geometry, property optimization, docking or target-based enrichment	Powerful data-driven paradigm; ImagiChem instead uses deterministic visual-to-chemical translation and does not require training
ImagiChem Library mode	Artistic image → row-level pixel statistics + global artistic profile → curated pharmacophore-core assembly	No	Curated Enamine single-pharmacophore cores plus rule-based visual mapping	Yes	Yes	Translate structured visual features into chemically valid molecules using pharmacophore-relevant curated cores	Validity, novelty, DBPP enrichment, SA, PAINS, noise control, IVS	Best suited to test whether structured artistic inputs enrich drug-like output relative to random noise
ImagiChem From-Scratch mode	Artistic image → global artistic profile → rule-based molecular blueprint	No	Rule-based chemical grammar; no Enamine core library	Yes	Yes	Generate molecules de novo from image-derived structural blueprints without using curated cores	Validity, DBPP, physicochemical constraints, mode-level comparison to noise	Demonstrates scaffold-library independence, although noise may outperform paintings in this mode because high-entropy input promotes broader graph exploration
ImagiChem Hybrid mode	Artistic image → combined Library + From-Scratch engines → merged unique SMILES output	No	Combination of curated pharmacophore cores and from-scratch rule-based grammar	Yes	Yes	Combine curated chemical realism with unconstrained structural creativity	Validity, novelty, DBPP enrichment, statistical painting-vs-noise validation, IVS	Recommended practical configuration; balances DBPP enrichment from artistic input with broader structural exploration

[a] The table is not intended as a quantitative benchmark, because ImagiChem addresses a different generative problem: deterministic translation of visual structure into chemical space rather than training-based reproduction of molecular data distributions.

Table S2. Drug-Likeness Distribution Across Artworks Based on DBPP Threshold (0.736) calculated on From Scratch mode.

Artwork	Total molecules	DBPP score >0.736	% Above Threshold
Bacchus	800	19	2.38
Bathing Women	800	15	1.88
Madonna of the Meadow	800	18	2.25
Mona Lisa	800	14	1.75
Starry Night	800	15	1.88
The Birth of Venus	800	14	1.75
The Scream	800	12	1.5
The Wedding at Cana	800	32	4
Virgin Annunciate	675	18	2.67
Vucciria	675	21	3.11
TOTAL	7750	178	2.3

Table S3. Drug-Likeness Distribution Across Artworks Based on DBPP Threshold (0.736) calculated on Library Only mode.

Artwork	Total molecules	DBPP score >0.736	% Above Threshold
Bacchus	1500	10	0.67
Bathing Women	1452	4	0.28
Madonna of the Meadow	4699	37	0.79
Mona Lisa	1800	48	2.67
Starry Night	1391	65	4.67
The Birth of Venus	1307	0	0
The Scream	1800	5	0.28
The Wedding at Cana	1718	4	0.23
Virgin Annunciate	675	18	2.67
Vucciria	675	4	0.59
TOTAL	17017	195	1.15

Table S4. Structural diversity and physicochemical profiles of ImagiChem-generated molecules across modes. Data includes total valid molecules, unique Bemis-Murcko scaffolds (Unique BM), and scaffold diversity (%). Internal structural diversity is calculated as 1 minus the mean Tanimoto similarity (Morgan fingerprints, radius = 2). Pharmacological evaluation reports mean Molecular Weight (MW), Topological Polar Surface Area (TPSA), Quantitative Estimate of Drug-likeness (QED), and Synthetic Accessibility (SA) scores, alongside the percentage of molecules passing Lipinski's Rule of Five, Veber's rules, and triggering PAINS alerts.

Mode[a]	Total Input	Unique BM	Scaffold Diversity (%)	Structural Diversity	Mean MW	Mean TPSA	Mean QED	Mean SA	Lipinski Pass (%)	Veber Pass (%)	PAINS Alerts (%)
H	18495	1971	10.66	0.87	336.4	56.41	0.67	4.35	100	88.92	0.98
LO	17017	222	1.3	0.84	333.25	48.32	0.67	4.43	100	83.8	0.21
FS	7750	2838	36.62	0.85	353.12	88.45	0.63	4.06	100	98.01	3.64

[a] H = Hybrid mode, LO = Library Only mode, FS = From Scratch mode.

Table S5. This Table Summarizes the Protein Models Utilized During the IVS (Protein) Phase. For Each Model, the Identification Code (PDB ID) is Provided, Along with the Source Database from which it was Obtained: the Protein Data Bank (PDB) or PDB-REDO (REDO). Additionally, the Spatial Coordinates Defining the Center of the Docking Simulation Box (Center x, Center y, Center z) are Listed, as are the Dimensions of the Simulation Box Along Each Axis (Npts x, Npts y, Npts z). A Uniform Spacing Value of 1 Å was Applied Across All Simulation Boxes to Maintain Consistency in Grid Resolution.

Protein	PDB ID	Source	Center x	Center y	Center z	Npts x	Npts y	Npts z
Adenosine A_{2A}	5OLH	REDO	−21.392	11.064	15.644	22	32	26
Tyrosine-protein kinase ABL2	2XYN (C)	PDB	−63.101	10.422	−34.090	20	22	18
AKT2	3D0E (A)	PDB	21.454	−20.504	8.109	30	26	26
Acetyl-CoA carboxylase 2	3JRX	PDB	−45.861	24.596	−18.365	22	24	24
Acetylcholinesterase	6O4W (B)	REDO	89.919	94.443	17.303	28	24	20
BACE-1	4DJW (B)	REDO	16.915	30.739	57.257	30	24	22
Carbonic Anhydrase II	4HT2 (A)	REDO	−3.826	24.016	−4.778	26	20	24
Cyclin-dependent kinase 2	1KE9	PDB	−9.642	48.089	9.126	22	20	24
Dopamine D3	7BVQ (B)	PDB	3.533	18.093	−15.119	20	26	24
Dopamine D4	5WIV	PDB	−17.723	15.366	−18.918	22	24	24
Epoxide hydrolase	3WKE	PDB	−16.428	−4.880	68.334	22	22	26
Estrogen receptor beta	4J24	PDB	−25.059	39.562	−15.778	40	30	40
FABP4	7FXP	PDB	5.808	−5.026	−18.748	20	26	20
HDAC6	5EDU (B)	REDO	0.053	9.322	5.058	24	22	20
HSP90α	4O09	REDO	2.893	−7.723	−24.333	22	20	24
Histamine H2	8YN3	PDB	96.425	113.687	73.330	20	20	24
Histamine H3	8YN5	PDB	83.635	127.608	125.033	24	22	22
MMP9	6ESM	PDB	1.365	50.745	19.807	24	20	22
Muscarinic M2	5YC8	PDB	182.291	26.539	523.962	24	20	26
Muscarinic M3	8E9W	PDB	132.204	126.493	157.463	20	22	24
Peroxisome proliferator-activated receptor gamma	4EMA	PDB	16.875	7.580	44.931	24	20	24
SIRT3	4JSR	REDO	26.037	46.249	−1.103	18	18	20
SYK	3EMG	REDO	17.324	40.930	9.178	22	30	26
α1A adrenergic	8THL	PDB	149.183	139.195	166.735	24	20	22
α1B adrenergic	7B6W	PDB	5.952	27.356	−11.503	24	20	24
β1 adrenergic	7BVQ	PDB	18.718	−23.726	87.090	26	24	32

Table S6. Comparative Performance Summary: Artistic Paintings *vs.* Random Noise across Generation Modes.

Mode	Input Type	Total Molecules	Hits (DBPP > 0.736)	Hit Rate (%)	Enrichment (P/RN)
Hybrid	Paintings (P)	18,495	243	1.31%	2.49×
	Random Noise (RN)	3,217	17	0.53%	
Library only	Paintings (P)	17,017	195	1.15%	11.46×
	Random Noise (RN)	4,000	4	0.10%	
From scratch	Paintings (P)	7,750	178	2.30%	0.37×
	Random Noise (RN)	549	34	6.19%	
